# Supplementary material for: An examination of gender imbalance in Scottish adolescents’ vocational interests
Source: PLoS One. 2021 Sep 24;16(9):e0257723. doi: 10.1371/journal.pone.0257723 (PMC8462744; doi:10.1371/journal.pone.0257723)
Supplement: Attachment — Submitted filename: PONE-D-21-04302 Review.docx [file pone.0257723.s001.docx]

PONE-D-21-04302

An Examination of Gender Imbalance in Scottish Adolescents' Vocational Interests

The manuscript is a descriptive study of RIASEC vocational interests among a sample of 1,306 Scottish students from 18 secondary schools. The study evaluates the structural validity, and the gendered distribution of the RIASEC scales items with a measure (SIMON-I) developed by Fonteyne et al. (2017) to assess students in the Flemish education system. In essence, the paper is a cross-cultural validation study of the SIMON-I.

Given that there is a relatively large literature in vocational psychology on the structural validity and gender distribution of Holland’s (1997) RIASEC personality types across cultural (country) samples, the issue with the present manuscript is how does this study contribute. There seems to be two contributions 1) the Forteyne et al. measure is relatively new and has to my knowledge not been evaluated in other countries; and 2) the RIASEC model has yet to be evaluated with a sample of Scottish students. These contributions fit the ongoing discussion of Holland’s model but do not add a variation on the model, extend the methodology of testing circular models, and add a new way to conceptualize interest models and cross-cultural assessment of Holland’s model. The gender differences among Scottish students are similar to findings from Su’s et al. (2009) meta-analysis of US samples. Recent research on the circular model have extended their studies to multiple cultures (nations)—see Glosenberg et al. (2019).

There are several methodological issues that can be addressed, beginning with the interpretation of the CFA.

1. The authors state on p. 16 that “Although most index values are of at least acceptable quality, the RMSEA values are too high.” They then dismiss the RMSEA as a fit measure because of degrees of freedom being small. The dismissal is premature, see Nagy et al. (2011) Table 3 and Table 4 and their explanation of the CFA for 7 different Holland models. Clearly, the CFA model tested by the current authors does not fit. I would add that it would strengthen the paper to test more than one variation of Holland’s model.
2. The footnote on page is inappropriate because you do not apply statical significant tests to the RIASEC intercorrelations since they are not independent. That is the reason the randomization test was introduced (Hubert & Arabie, 1987).
3. Interpretation of benchmarks and the p-value of the randomization test is incorrect. On page 17 the authors state, “All our correspondence indices (CI) are well above the international benchmarks (0.48) suggested by Rounds and Tracey (1996), while a circular fit of the data reached a significance of at least p = 0.05.” Rounds and Tracey reported that the mean CI was .48 for international matrices, almost 3 SDs below US mean CI. Most of the international samples did not support a circular structure. The correct interpretation of the p-value is: Given the circular model of RIASEC, statistically significant p-value indicates that random ordering of the RIASEC types can be rejected.
4. Over interpretation of the structural tests. On page 18, the authors state, “In summary, the circular fit for our data has been confirmed by both CFA and RTOR analyses. As a consequence, the use of the Flemish instrument is suited for cross-cultural application to a Scottish context.” Structural tests are necessary but not sufficient for application of measures cross-culturally.

Alexander Glosenberg, Terence J.G. Tracey, Tara S. Behrend, David L. Blustein, Lori L. Foster, (2019). Person-vocation fit across the world of work: Evaluating the generalizability of the circular model of vocational interests and social cognitive career theory across 74 countries,

Journal of Vocational Behavior, 112, 92-108.

Hubert, L., & Arabie, P. (1987). Evaluating order hypotheses with proximity matrices. Psychological Bulletin, 102, 172-178.

Nagy, G., U. Trautwein, U., & Lüdtke, O. (2010). The structure of vocational interests in Germany: Different methodologies, different conclusions. Journal of Vocational Behavior, pp. 153-169.
